# Supplementary material for: Causal Relationship between Aging and Anorexia Nervosa: A White-Matter-Microstructure-Mediated Mendelian Randomization Analysis
Source: Biomedicines. 2024 Aug 16;12(8):1874. doi: 10.3390/biomedicines12081874 (PMC11351342; doi:10.3390/biomedicines12081874)
Supplement: Supplementary file 1 [file biomedicines-12-01874-s001.zip › Supplementary Figure/Figure S4/Singe SNP plot of MR analysis for TL to ubm-b-2071.pdf]

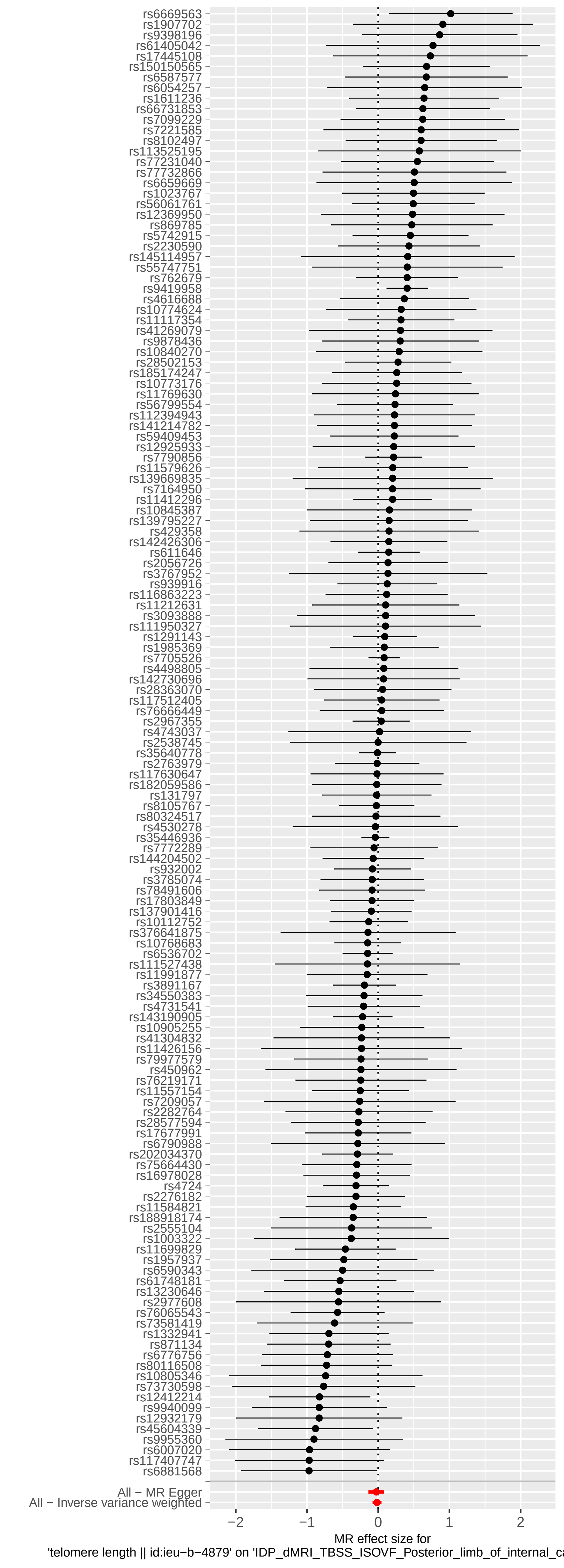
rs6669563
rs1907702
rs9398196
rs61405042
rs17445108
rs150150565
rs6587577
rs6054257
rs1611236
rs66731853
rs7099229
rs7221585
rs8102497
rs113525195
rs77231040
rs77732866
rs6659669
rs1023767
rs56061761
rs12369950
rs869785
rs5742915
rs2230590
rs145114957
rs55747751
rs762679
rs9419958
rs4616688
rs10774624
rs11117354
rs41269079
rs9878436
rs10840270
rs28502153
rs185174247
rs10773176
rs11769630
rs56799554
rs112394943
rs141214782
rs59409453
rs12925933
rs7790856
rs11579626
rs139669835
rs7164950
rs11412296
rs10845387
rs139795227
rs429358
rs142426306
rs611646
rs2056726
rs3767952
rs939916
rs116863223
rs11212631
rs3093888
rs111950327
rs1291143
rs1985369
rs7705526
rs4498805
rs142730696
rs28363070
rs117512405
rs76666449
rs2967355
rs4743037
rs2538745
rs35640778
rs2763979
rs117630647
rs182059586
rs131797
rs8105767
rs80324517
rs4530278
rs35446936
rs7772289
rs144204502
rs932002
rs3785074
rs78491606
rs17803849
rs137901416
rs10112752
rs376641875
rs10768683
rs6536702
rs111527438
rs11991877
rs3891167
rs34550383
rs4731541
rs143190905
rs10905255
rs41304832
rs11426156
rs79977579
rs450962
rs76219171
rs11557154
rs7209057
rs2282764
rs28577594
rs17677991
rs6790988
rs202034370
rs75664430
rs16978028
rs4724
rs2276182
rs11584821
rs188918174
rs2555104
rs1003322
rs11699829
rs1957937
rs6590343
rs61748181
rs13230646
rs2977608
rs76065543
rs73581419
rs1332941
rs871134
rs6776756
rs80116508
rs10805346
rs73730598
rs12412214
rs9940099
rs12932179
rs45604339
rs9955360
rs6007020
rs117407747
rs6881568
All − MR Egger
All − Inverse variance weighted
−2
−1
0
1
2
MR effect size for
'telomere length || id:ieu−b−4879' on 'IDP_dMRI_TBSS_ISOVF_Posterior_limb_of_internal_ca
